# Polygenic analysis and targeted improvement of the complex trait of high acetic acid tolerance in the yeast *Saccharomyces cerevisiae*

**DOI:** 10.1186/s13068-015-0421-x

**Published:** 2016-01-06

**Authors:** Jean-Paul Meijnen, Paola Randazzo, María R. Foulquié-Moreno, Joost van den Brink, Paul Vandecruys, Marija Stojiljkovic, Françoise Dumortier, Polona Zalar, Teun Boekhout, Nina Gunde-Cimerman, Janez Kokošar, Miha Štajdohar, Tomaž Curk, Uroš Petrovič, Johan M. Thevelein

**Affiliations:** Laboratory of Molecular Cell Biology, Institute of Botany and Microbiology, KU Leuven, Leuven-Heverlee, Belgium; Department of Molecular Microbiology, VIB, Kasteelpark Arenberg 31, Flanders, 3001 Leuven-Heverlee, Belgium; CBS, Fungal Biodiversity Centre (CBS-KNAW), Utrecht, The Netherlands; Department of Biology, Biotechnical Faculty, University of Ljubljana, Večna pot 111, 1000 Ljubljana, Slovenia; Centre of Excellence for Integrated Approaches in Chemistry and Biology of Proteins, Jamova 39, 1000 Ljubljana, Slovenia; Genialis d.o.o., Ulica Zore Majcnove 4, 1000 Ljubljana, Slovenia; Faculty of Computer and Information Science, University of Ljubljana, Večna pot 113, Ljubljana, Slovenia; Department of Molecular and Biomedical Sciences, Jožef Stefan Institute, Jamova 39, Ljubljana, Slovenia

**Keywords:** Bioethanol production, Acetic acid tolerance, Polygenic analysis, QTL mapping, Pooled-segregant whole-genome sequence analysis, Inbreeding, *Saccharomyces cerevisiae*

## Abstract

**Background:**

Acetic acid is one of the major inhibitors in lignocellulose hydrolysates used for the production of second-generation bioethanol. Although several genes have been identified in laboratory yeast strains that are required for tolerance to acetic acid, the genetic basis of the high acetic acid tolerance naturally present in some *Saccharomyces cerevisiae* strains is unknown. Identification of its polygenic basis may allow improvement of acetic acid tolerance in yeast strains used for second-generation bioethanol production by precise genome editing, minimizing the risk of negatively affecting other industrially important properties of the yeast.

**Results:**

Haploid segregants of a strain with unusually high acetic acid tolerance and a reference industrial strain were used as superior and inferior parent strain, respectively. After crossing of the parent strains, QTL mapping using the SNP variant frequency determined by pooled-segregant whole-genome sequence analysis revealed two major QTLs. All F1 segregants were then submitted to multiple rounds of random inbreeding and the superior F7 segregants were submitted to the same analysis, further refined by sequencing of individual segregants and bioinformatics analysis taking into account the relative acetic acid tolerance of the segregants. This resulted in disappearance in the QTL mapping with the F7 segregants of a major F1 QTL, in which we identified *HAA1*, a known regulator of high acetic acid tolerance, as a true causative allele. Novel genes determining high acetic acid tolerance, *GLO1, DOT5*, *CUP2*, and a previously identified component, *VMA7*, were identified as causative alleles in the second major F1 QTL and in three newly appearing F7 QTLs, respectively. The superior *HAA1* allele contained a unique single point mutation that significantly improved acetic acid tolerance under industrially relevant conditions when inserted into an industrial yeast strain for second-generation bioethanol production.

**Conclusions:**

This work reveals the polygenic basis of high acetic acid tolerance in *S. cerevisiae* in unprecedented detail. It also shows for the first time that a single strain can harbor different sets of causative genes able to establish the same polygenic trait. The superior alleles identified can be used successfully for improvement of acetic acid tolerance in industrial yeast strains.

## Background

Acetic acid tolerance in yeast is a trait of high industrial importance since yeast fermentation is severely inhibited by low levels of this weak organic acid. The presence of high acetic acid levels in lignocellulosic hydrolysates strongly reduces the fermentative capacity of yeast [1–5]. Especially, the artificially engineered capacity of pentose fermentation suffers from the presence of acetic acid [1, 6, 7], emphasizing the importance of high acetic acid tolerance to enable efficient conversion of all sugars in lignocellulosic hydrolysates to ethanol. Multiple attempts to rationally engineer increased acetic acid tolerance in yeast have met with limited success, possibly because a high number of genes appears to be involved in the response to acetic acid stress and in establishing high intrinsic acetic acid tolerance [8–13]. Random approaches, such as evolutionary adaptation, have resulted in strains with improved acetic acid tolerance [14, 15], but this method of focused selection on a single trait often leads to loss of other important properties in industrial yeast strains.

A central challenge in modern biology is to understand the interplay of genes, proteins, and other components that determine complex physiological properties like high acetic acid tolerance. In the past, research focussed primarily on the identification of single alleles or genetic loci that are involved in physiological traits [16]. However, in contrast to Mendelian traits (traits that are caused by a single locus), quantitative traits are established by multiple interacting genetic loci, which makes elucidation of their genetic basis much more difficult [17]. In addition, the genetic mapping of quantitative trait loci (QTL) is hampered by genetic heterogeneity, variable phenotypic contributions of each QTL, epistasis and gene-environment interactions [18]. These limitations have stimulated the development of novel technologies to simultaneously identify genomic loci that are involved in complex traits. With these technologies, phenotypes like high-temperature tolerance, efficient sporulation, and chemical resistance have been genetically unraveled [17, 19, 20].

Recently, our lab has developed a strategy, called pooled-segregant whole-genome sequence analysis, that allows simultaneous mapping of QTLs underlying a complex trait using small populations of segregants [21]. This is particularly important for complex traits that require elaborate experimental work to score. This technology has been employed successfully to identify genetic determinants that are involved in high ethanol tolerance of cell proliferation [21], maximal ethanol accumulation capacity [22], low glycerol production [23, 24], and high thermotolerance [25] in the yeast *Saccharomyces cerevisiae*. Identification of the causative alleles in the QTLs was accomplished using reciprocal hemizygosity analysis (RHA) [17]. However, pinpointing causative mutations in the QTLs remains laborious since this method results in QTLs still containing a relatively large number (20-50) of candidate causative genes in the center of the locus. Reduction of QTL size can be achieved by increasing the recombination frequency through inbreeding and selecting millions of segregants, as was described by Parts et al. [26]. However, the use of very large pools makes it cumbersome for analyzing industrially relevant traits, which in addition are often not directly selectable. Furthermore, although inbreeding crosses can be used to decrease the size of QTLs, it remains unknown whether it can also influence the number and nature of QTLs, especially in mapping of minor loci.

In the present paper, we have applied the polygenic analysis platform to elucidate the genetic basis of high acetic acid tolerance. We have crossed a strain with high acetic acid tolerance with an industrial reference strain and we have used both F1 segregants and F7 segregants, obtained after multiple inbreeding of all F1 segregants, for QTL mapping with pooled-segregant whole-genome sequence analysis. We show that the increased recombination frequency in the F7 segregants results in reduced QTL size, facilitating the identification of causative genes, but unexpectedly also in appearance of new QTLs and disappearance of previously validated QTLs, compared to QTL mapping with F1 segregants. Furthermore, by sequencing the individual segregants of the F7 pool, combined with bioinformatics and statistical analysis, we were able to map QTLs close to single gene level. In this way, we have elucidated the genetic basis of high acetic acid tolerance in *S. cerevisiae* with an unprecedented level of detail, identifying *HAA1*, a known regulator of high acetic acid tolerance, and *GLO1, DOT5*, *CUP2*, and *VMA7* as novel genes determining high acetic acid tolerance.

## Results

### Screening for superior acetic acid tolerance

**Fig. 1:**
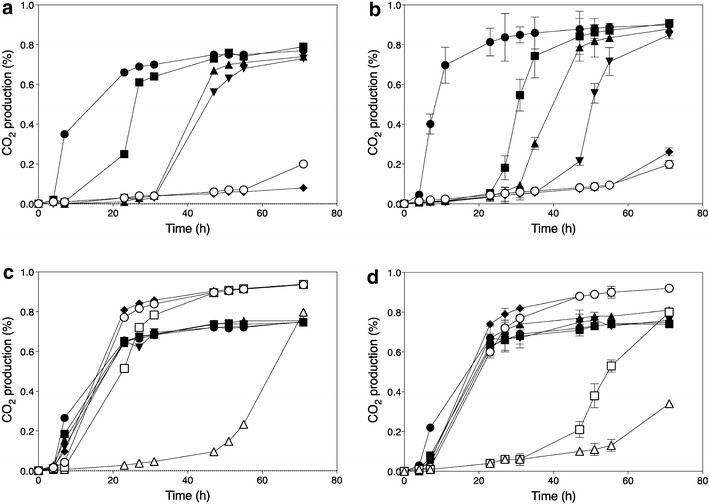
Fermentation profiles in the presence of different concentrations of acetic acid. CO_2_ production was determined from the weight loss during fermentation and expressed as percentage of initial weight of the total culture medium. **a** Acetic acid sensitive diploid strain Ethanol Red. **b** Acetic acid sensitive Ethanol Red haploid segregant ER18. **c** Acetic acid-tolerant diploid strain JT22689. **d** Acetic acid-tolerant JT22689 haploid segregant 16D. Strains were inoculated in YPD medium with 2 % glucose at pH 4 and various concentrations of acetic acid: 0 % (), 0.4 % (v/v) (), 0.5 % (v/v) (), 0.6 % (v/v) (), 0.7 % (v/v) (), 0.8 % (v/v) (), 0.9 % (v/v) (), 1.0 % (v/v) (). Data points are the average of duplicate measurements; *error bars* represent the maximum deviation of the average

Ethanol Red is a diploid industrial yeast strain that is used world-wide for commercial first-generation bioethanol production and has also been used as a platform strain for the development of an industrial xylose-fermenting strain for second-generation bioethanol production [27]. It is able to produce ethanol titers of up to 18 %. The fermentation performance of this strain is severely affected by acetic acid, a weak organic acid present in high quantities in lignocellulose hydrolysates and other industrial fermentation media. In semi-anaerobic, static small-scale fermentations, Ethanol Red could still ferment glucose in the presence of 0.6 % (v/v) acetic acid in YPD medium at pH 4.0. However, the lag phase was strongly prolonged, from about 5 h in the absence of acetic acid to approximately 30–40 h in the presence of 0.5–0.6 % (v/v) acetic acid (Fig. 1a). To determine the polygenic basis of high acetic acid tolerance, we have used Ethanol Red as the inferior parent strain displaying lower acetic acid tolerance. For that purpose, we sporulated Ethanol Red and selected the haploid segregant, ER18, which showed similar acetic acid tolerance as Ethanol Red (Fig. 1b).

To identify an *S. cerevisiae* strain with very high acetic acid tolerance, we have screened the MCB (KU Leuven) strain collection, the strain collection from the CBS Fungal Biodiversity Centre (CBS-KNAW, Utrecht, The Netherlands) and Ex Culture Collection of the Infrastructural Centre Mycosmo, MRIC UL, Slovenia (http://www.ex-genebank.com/), at the Department of Biology at the University of Ljubljana. The Ljubljana collection included 141 newly isolated strains from diverse habitats, including spoilt vinegar. First, we performed a very stringent screen for growth on solid YPD medium in the presence of 0.95 % acetic acid at pH 4.0 in order to pre-select candidate strains to be tested subsequently in small-scale fermentations. In total, more than 1000 *S. cerevisiae* strains were evaluated in this way and only 9 strains were able to grow under these conditions. They were subsequently evaluated in semi-anaerobic, small-scale fermentations with YPD medium (pH 4.0) in the presence of 0.7 % (v/v) acetic acid and higher concentrations in repetitions with the best strains. Strain JT22689 (PYCC 4542), which was originally isolated from fermenting must (‘sturm’) in Austria, showed the best performance in the presence of high acetic acid concentrations, being able to ferment glucose in the presence of 0.8 % (v/v) acetic acid without a lag phase and with a similar rate as in the absence of acetic acid (Fig. 1c). A haploid segregant, 16D, with similarly high acetic acid tolerance, was isolated from strain JT22689 in order to perform the genetic mapping (Fig. 1d).

### QTL mapping with pooled F1 segregants

Mapping the genetic determinants that are responsible for high acetic acid tolerance was initiated by crossing the superior segregant 16D with the inferior segregant ER18 and sporulating the diploid hybrid strain. The segregants of the hybrid were screened for high acetic acid tolerance of fermentation. This resulted in the identification of 27 out of 288 segregants that were able to ferment glucose in the presence of 0.9 % (v/v) acetic acid with a similar rate as that of the superior parent strain 16D. These 27 segregants were selected for pooled-segregant whole-genome sequence analysis. Genomic DNA isolated from the two parent strains, from the pool of 27 selected segregants, and from a control pool of 27 randomly selected segregants was sent for custom sequence analysis with the Illumina HiSeq 2000 technology (BGI, Hong Kong). The sequence reads from parent strains 16D and ER18 were aligned with the reference sequence of strain S288C. A total number of 23,150 single nucleotide polymorphisms (SNPs) between 16D and ER18 was found, which were subsequently quality filtered as previously described [28]. The SNP variant frequency was calculated as the percentage of the variant from the superior parent strain on the total number of aligned reads and expressed as a figure between 0 and 1. The SNP variant frequency was subsequently plotted against the respective chromosomal position of the SNP. The underlying structure in the SNP variant frequency scatter plot of a given chromosome was identified by fitting smoothing splines in the generalized linear mixed model framework, as described previously [29]. Variant frequencies that significantly deviate from 0.5 (which corresponds with random segregation) are indicative of genetic linkage with the phenotype, either linked to the genome of the superior parent (>0.5) or to that of the inferior parent (<0.5).

**Fig. 2:**
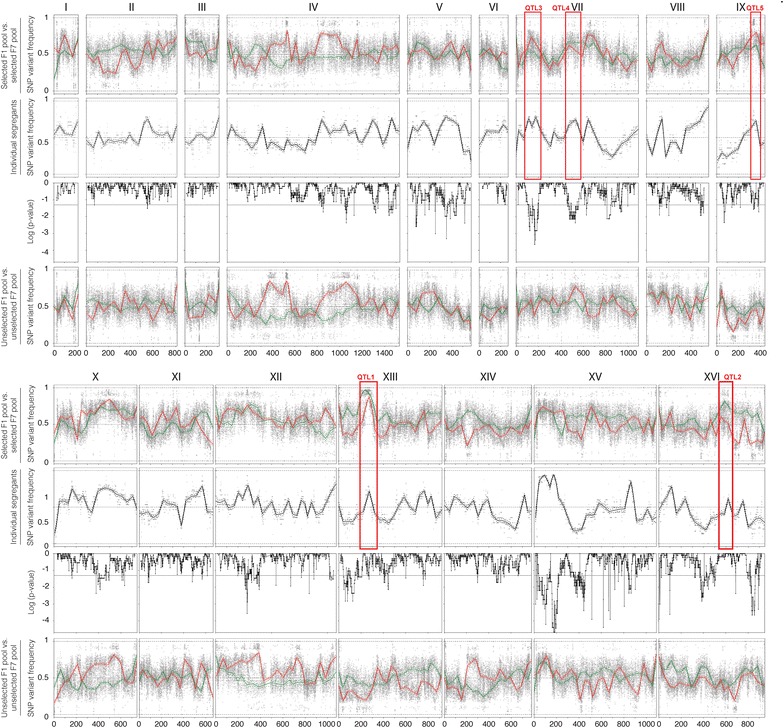
QTL mapping of high acetic acid tolerance. The mapping was performed with pooled F1 segregants (*green*), pooled F7 segregants (*red*), and individual F7 segregants (*black*, *second row*). Pooled F1 and pooled F7 segregants (27 segregants for both pools) were subjected to sequence analysis with the Illumina platform at BGI. Individual F7 segregants were sequenced with the Illumina platform at EMBL. P values calculated using the individual sequencing data from F7 segregants were plotted against the respective chromosomal position (*third row*). *p* values <0.05 (indicated by *dotted line*) were considered statistically significant. Unselected pools consisting of 27 randomly selected segregants were also sequenced to eliminate linkage to inadvertently selected traits (*bottom row*, F1 segregants: *green*, F7 segregants: *red*)

**Fig. 3:**
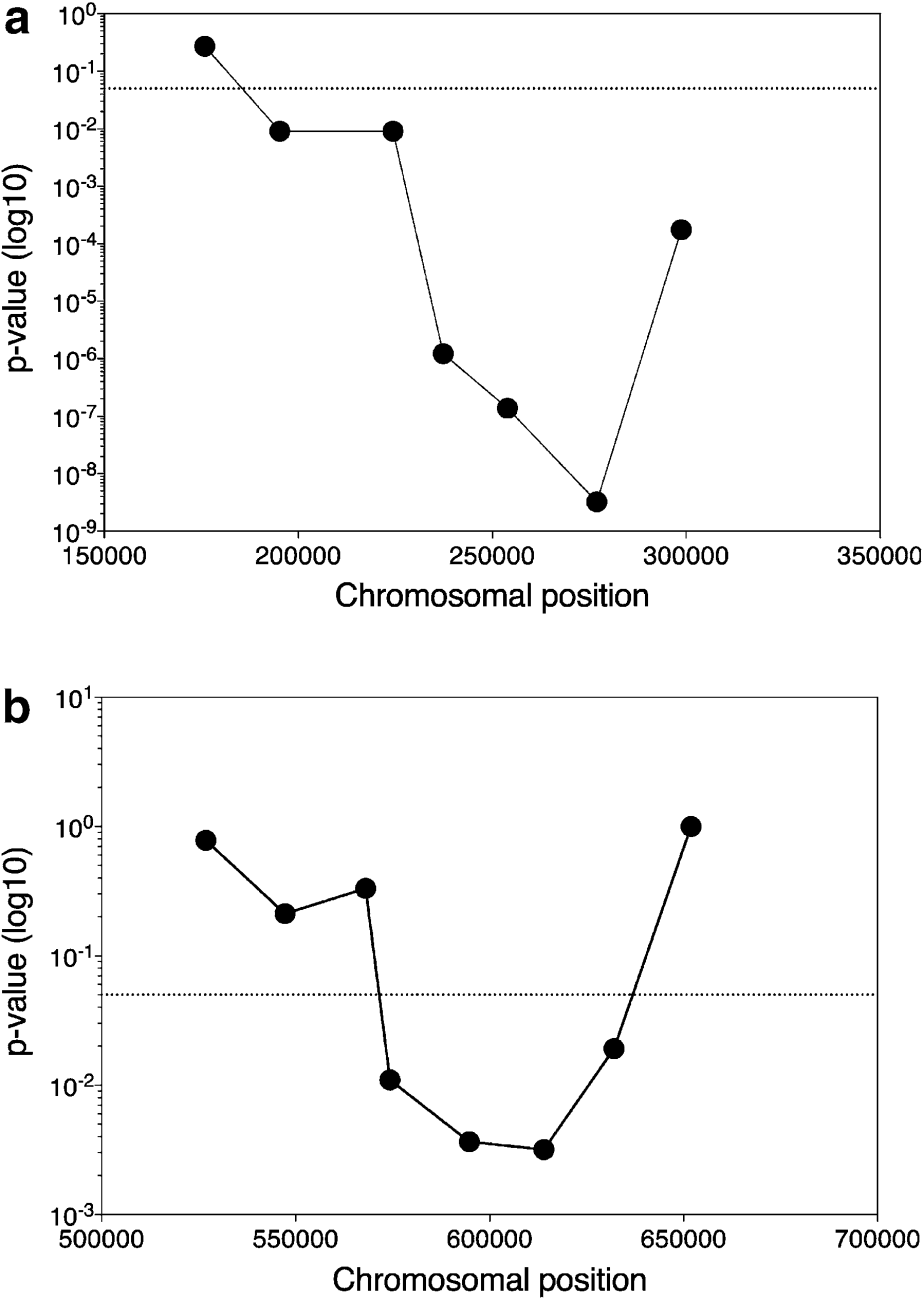
QTL mapping with selected SNPs in the individual segregants. Selected SNPs in QTL1 (**a**) and QTL2 (**b**) were scored with allele-specific PCR, the SNP variant frequency and the corresponding *p* values were calculated, and the *p* values were plotted over the length of the chromosomes, XIII for QTL1 (**a**) and XVI for QTL2 (**b**)

The results of the QTL mapping with the pooled F1 segregants (Fig. 2, green lines) reveal two loci with a strong linkage to the genome of the superior segregant 16D: QTL1 on chromosome XIII and QTL 2 on chromosome XVI. The statistical significance of QTL1, located on chromosome XIII between position 181,019 and 294,166, was confirmed using a hidden Markov model (HMM) [30]. Although the HMM did not show statistical significance for QTL2, the SNP variant frequency was such that we decided to study both QTLs in further detail. To this end, selected SNPs in the 27 individual segregants were scored by allele-specific PCR to determine the SNP variant frequency and the statistical significance of the genetic linkage precisely. When the *p* values for the selected SNPs were plotted against the corresponding position on the chromosomes (XIII and XVI), two QTLs were revealed (Fig. 3). Using a binomial test previously described [21, 29], both loci were found to be statistically significant. Furthermore, the size of the QTLs could be downscaled to the regions 194,000–277,000 bp for QTL1 on chromosome XIII, and 568,000–615,000 bp for QTL2 on chromosome XVI, with the center of the loci further confined to much smaller regions (Fig. 3).

### Identification of causative genes by RHA and allele exchange in F1 QTLs

**Fig. 4:**
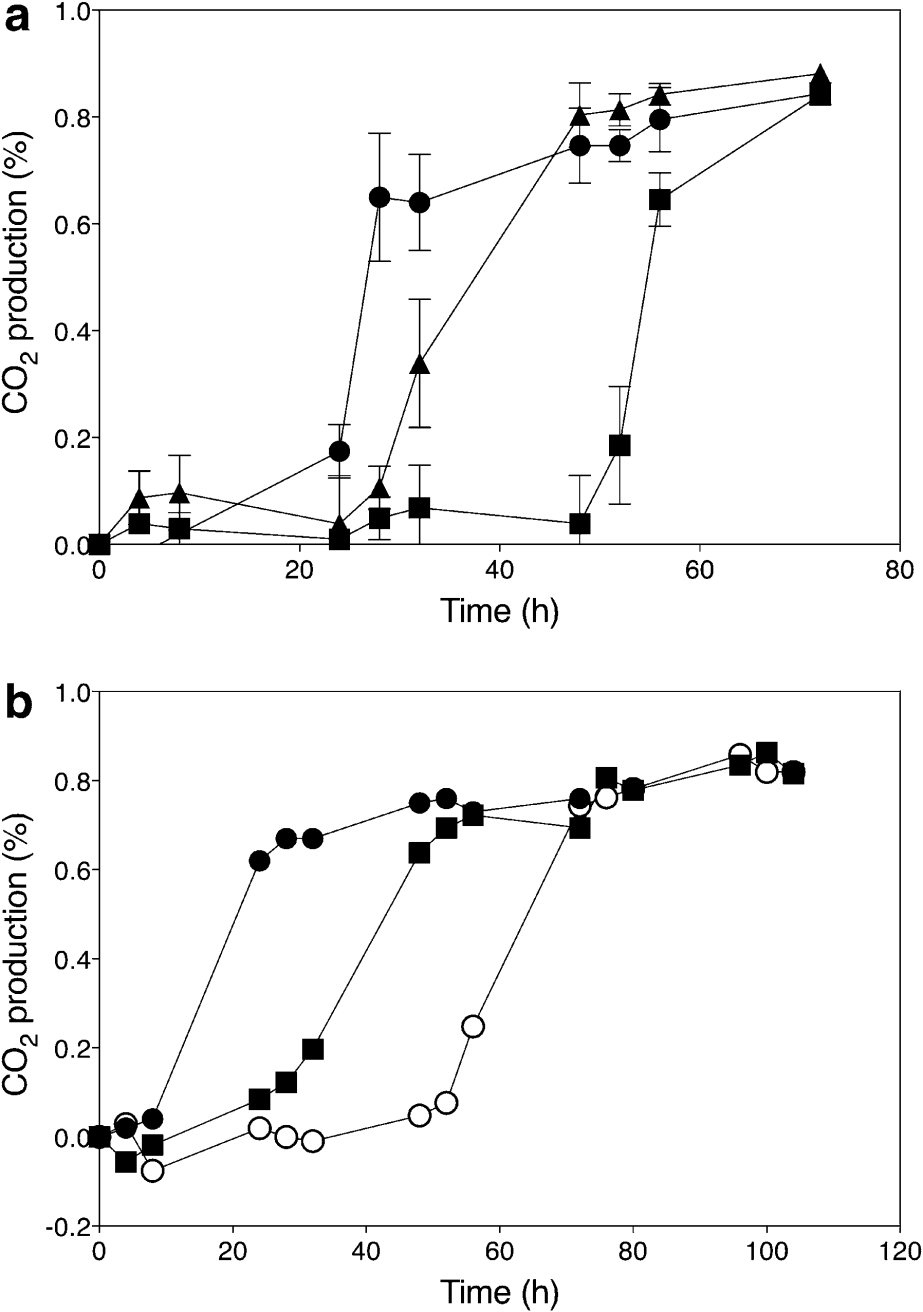
Identification of *HAA1* as the causative allele in QTL2 on Chr. XVI. **a** Fermentation profiles of the hemizygous diploid strains used in the RHA for *HAA1*. Fermentations were performed in YPD medium with 2 % glucose and supplemented with 0.7 % (v/v) acetic acid at pH 4.0. Three diploid hybrid strains were tested and compared: ER18 *haa1*Δ x 16D (); ER18 × 16D *haa1*Δ (); ER18 × 16D (). Data points are the average of duplicate measurements. *Error bars* represent the maximum deviation of the average. **b**. Fermentation profiles of the strains 16D (), ER18 (), and ER18-HAA1 () (ER18 in which the complete *HAA1* gene with promoter, ORF and terminator, was replaced by the *HAA1* allele from 16D) in YPD medium with 2 % glucose and supplemented with 0.6 % (v/v) acetic acid at pH 4.0

For further analysis of the two identified QTLs, genes located within the center of the linked regions were identified with the *Saccharomyces* genome database. In QTL1, none of the genes present had previously been linked to acetic acid tolerance. The causative gene in QTL1 was identified later using the F7 segregants (see further). On the other hand, the gene *HAA1* located in QTL2 is a well-known determinant of acetic acid tolerance. *HAA1* encodes a transcriptional activator involved in adaptation to weak acid stress [10, 31]. This gene was therefore further evaluated for possible causative character using the RHA method [17]. Two hemizygous diploid 16D/ER18 hybrid strains were constructed, which retained a single copy of the *HAA1* allele either from the superior or inferior parent, while the other copy of the gene was deleted. When these RHA strains were tested in fermentations with acetic acid, a clear difference was observed between the reciprocal strains. The *HAA1* allele from the superior parent strain 16D sustained a much faster fermentation rate than the allele from the inferior parent strain ER18 (Fig. 4a). In addition, a strain was constructed by replacing the whole *HAA1* allele (promoter + ORF + terminator) in strain ER18 with the allele from 16D. Semi-anaerobic static fermentations with YPD2 % performed with this strain in the presence of different concentrations of acetic acid showed that acetic acid tolerance was clearly improved (Fig. 4b and results not shown). The improvement, however, was only partial confirming the polygenic nature of high acetic acid tolerance. These results identified *HAA1* as a causative allele in QTL2.

### QTL mapping with pooled F7 segregants

The QTLs identified with the F1 pool were relatively large in size. Therefore, we attempted to narrow down the QTLs by inbreeding all F1 segregants multiple times. After six rounds of inbreeding (for experimental details on inbreeding crosses, see Sect. “Methods”), the F7 segregants were screened for high acetic acid tolerance under the same conditions used for screening the F1 segregants. Out of 768 segregants assessed, 66 segregants showed a high fermentation rate in the presence of 0.9 % (v/v) acetic acid. For statistical reasons, we decided to use a pool of similar size as the F1 pool to perform the pooled-segregant whole-genome sequencing analysis, i.e., we used a pool of the 27 best segregants. In addition, a pool of 27 unselected F7 segregants was sequenced in order to discriminate between truly linked QTLs and inadvertently selected QTLs due to the inbreeding crosses strategy.

The QTL mapping results obtained with the pool of F7 segregants were compared with those obtained with the pool of F1 segregants (Fig. 2). We expected the mapped QTLs to become narrower in size due to the increase in recombination frequency. This was indeed observed in specific cases. For instance, the size of QTL1 on chromosome XIII obtained with the F7 segregants had been reduced to approximately 30 kb (position 247,466–277,019), which is about 83 kb smaller than the QTL1 obtained with the F1 segregants. Moreover, the number of genes in the center of the locus dramatically decreased to only 6, (*GIS4*, *TRM12*, *GLO1*, *YML002W*, *YML003W*, *YPT7*) which strongly facilitated identification of the causative gene (see further).

On the other hand, the increase in recombination frequency also resulted in several unexpected outcomes. The QTL2 mapped with the F1 segregants on chr. XVI, for which we identified *HAA1* as the causative gene with RHA analysis, surprisingly was no longer present (Fig. 2). Its absence was confirmed with the mapping based on SNP analysis in the individual F7 segregants. This indicates that the F7 segregants no longer needed the superior *HAA1* allele from the superior parent strain 16D to display high acetic acid tolerance. Apparently, the polygenic phenotype of high acetic acid tolerance was now supported by another, partially different set of causative genes. This conclusion was supported by the appearance of several new QTLs in the mapping with the F7 segregants, which were not yet present in the mapping with the F1 segregants. Two new QTLs were located on chromosome VII (QTL3 and 4) and one on chromosome IX (QTL5).

### QTL mapping with individually sequenced F7 segregants

In an attempt to further enhance the resolution of the QTL mapping, similar to what has been previously reported [32], we sequenced the 27 selected segregants from the F7 pool individually. Genomic DNA samples were sent to the Genomic Core Facility of EMBL (Heidelberg, Germany) and the sequencing data were treated with the previously used scripts that were modified for this purpose [30]. The main advantage of this approach is that the whole-genome sequence of the 27 selected segregants can be compared with each other, rather than only the individual reads. Hence, the SNP variant frequencies can now be calculated precisely for all SNPs using the whole-genome sequence of the individual segregants, instead of estimating the frequencies from the pooled sequencing reads. Furthermore, by aligning the 27 whole-genome sequences, we could score all SNPs along the genome in all single segregants and calculate the statistical significance of every single SNP, using the binomial test described previously [21, 29].

Figure 2 shows that the same QTLs could be identified using sequencing data from either the pooled segregants or the individual segregants. Thus, sequencing individual segregants yields comparable genetic maps as sequencing pooled segregants. However, additional information could be gained from the calculated *p* values. SNPs were considered statistically significant if the *p* value is lower than 0.05, and by combining the genetic mapping with the calculated *p* values, additional regions could be identified that might contain causative genes for acetic acid tolerance. We noticed that the inbreeding also caused appearance of conspicuous QTLs in the mapping with the pool of unselected F7 segregants, likely indicating linkage with inadvertently selected traits like sporulation and spore germination capacity. After eliminating these inadvertently linked regions and using this approach, we identified multiple regions in the genome that were statistically linked to the genome of the superior parent: QTL1 on chromosome XIII (position 261,255–271,498), QTL3 on chromosome VII (position 107,986–195,096), QTL4 on chromosome VII (position 471,171–554,980), and QTL5 on chromosome IX (position 335,344–340,345) (Fig. 2).

**Fig. 5:**
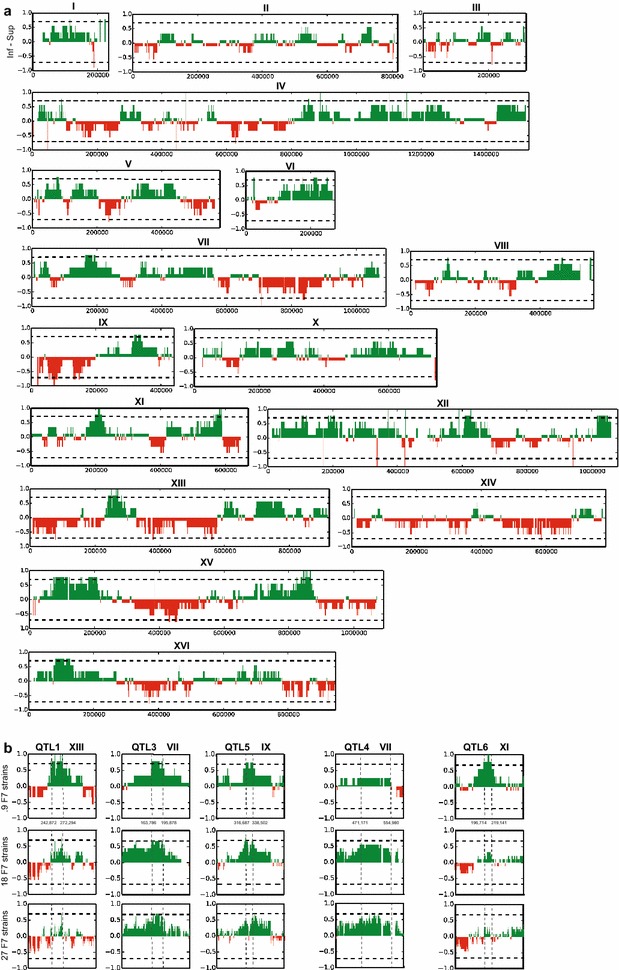
QTL mapping of high acetic acid tolerance using individual F7 segregants. **a** For each of the individual F7 segregants, the parent-of-origin linkage information was determined by using a distance-based method followed by the segmentation of individual chromosomes. Genomic regions linked either to the genome of the superior parent (>0; *green*) or to that of the inferior parent (<0; *red*) were derived by averaging the parent-of-origin linkage information for the nine most tolerant segregants of the F7 pool. *Horizontal lines* are included to mark the threshold values of ±0.7 used for QTL identification (see “Methods” for details). **b** Detailed view of the identified QTL regions when different numbers of F7 strains (the 9, 18, or 27 strains with highest acetic acid tolerance) are considered. Inclusion of additional strains with less extreme acetic acid tolerance results in QTL regions becoming either less pronounced (QTL1, QTL3, QTL5), emerge when less tolerant strains are considered (QTL4), or are present when only the most tolerant strains are included in the analysis (QTL6)

Further analysis of the SNP polymorphisms in the individual F7 segregants was carried out using bioinformatics analysis that takes the extent of the acetic acid tolerance of the individual segregants into account. Figure 5a shows the genome-wide genetic map obtained by averaging genomic linkage information over the nine most tolerant F7 segregants. These segregants were selected because their tolerance was higher than or comparable to that of the superior parent strain. By using this approach, several QTL regions were re-mapped: QTL1 on chromosome XIII (position 240,872–272,649) with *GLO1* as the causal gene, QTL3 on chromosome VII (position 163,768–195,878) with *CUP2* as the causal gene, and QTL5 on chromosome IX (position 316,687–338,502) with *DOT5* as the causal gene. Inclusion of additional F7 segregants in decreasing order with respect to acetic acid tolerance resulted in the loss of QTL detection ability/resolution, and emergence of previously non-detected QTLs (Fig. 5b), indicating the presence of different combinations of causative alleles in segregants with less extreme tolerance to acetic acid. Namely, while QTLs 1, 3, and 5 became less pronounced and/or distinct, QTL4 (comprising the *VMA7* causative gene) emerged only when less tolerant segregants were taken into account. Using this approach of taking into account the quantitative phenotype of the segregants actually resulted in identifying another genome region enriched for the superior parent SNPs that is specific for high acetic acid tolerance, QTL6 on chromosome XI (position 196,714–219,141). This type of analysis can thus pinpoint specific combinations of causative alleles.

### Identification of causative genes by RHA in F7 QTLs

**Fig. 6:**
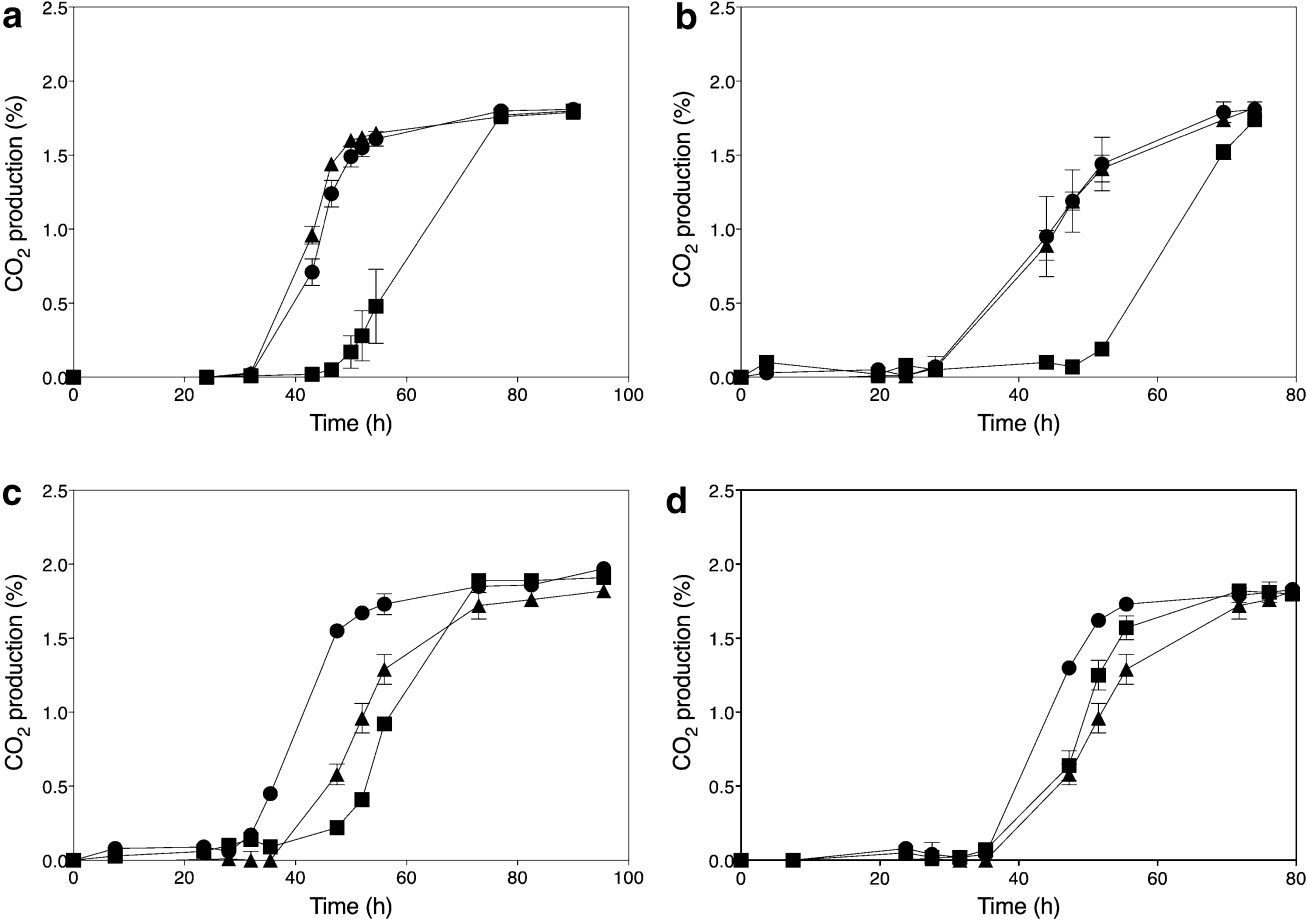
Fermentation profiles of the hemizygous diploid strains used in the RHA for *GLO1* (**a**), *CUP2* (**b**)*, DOT5* (**c**), and *VMA7* (**d**). CO_2_ production was determined from the weight loss during fermentation and expressed as percentage of initial weight of the total culture medium. Fermentations were performed in YPD [4 % glucose (w/v)] medium supplemented with 0.8 % (v/v) acetic acid at pH 4.0. Three diploid hybrid strains were tested and compared: ER18*gene*Δ × 16D (); ER18 × 16D*gene*∆ (); ER18 × 16D (). Data points are the average of duplicate measurements. *Error bars* represent the maximum deviation of the average

After identifying QTLs with the F7 segregants, a number of candidate genes was selected based on the statistical linkage to the phenotype predicted by the p values and the function according to the SGD database. The selected candidate genes were *YPT7* and *GLO1* (QTL1), *TOS3* and *CUP2* (QTL3), *PMA1* and *VMA7* (QTL4), and *DOT5* (QTL5). These genes were subsequently tested by the RHA method. After constructing the hemizygous diploid strains, fermentation experiments were performed in YPD medium with 0.8 % (v/v) acetic acid at pH 4.0 to assess the effect of the candidate genes on high acetic acid tolerance. The results of these fermentations, shown in Fig. 6, indicated that *GLO1*, *VMA7*, *DOT5*, and *CUP2* act as causative genes in high acetic acid tolerance of the superior haploid parent 16D. Furthermore, these results indicate that combining the mapping of the SNP variant frequency with calculation of the statistical significance of every SNP, using the whole-genome sequences of the individual segregants, strongly improves the resolution of QTL mapping.

### Natural occurrence of the causative alleles in other yeast strains

**Fig. 7:**
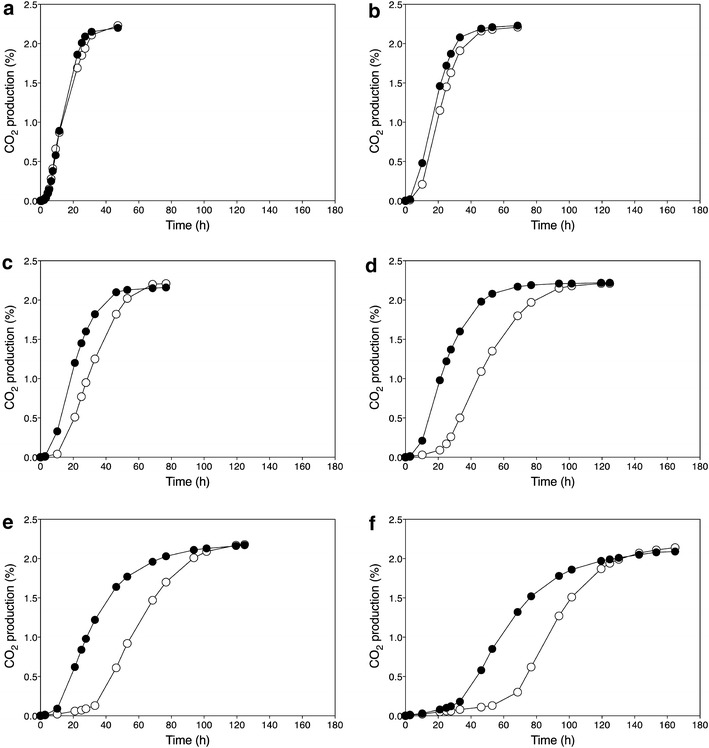
Fermentation profiles of the diploid strains GSE16-T18 and GSE16-T18-HAA1* (carrying the unique HAA1* mutation, changing G to A at position c.1517, of strain 16D in both *HAA1* alleles). Semi-anaerobic static fermentations were performed in YP medium with 20 % glucose at pH 5.2 and varying concentrations of acetic acid. Strains: GSE16-T18 () and GSE16-T18-HAA1* (). **a** No acetic acid; **b** 1.0 % acetic acid; **c** 1.2 % acetic acid; **d** 1.4 % acetic acid; **e** 1.6 % acetic acid; **f** 2.0 % acetic acid

The sequences of the causative genes identified with the F7 segregants were compared with the corresponding gene sequences in 28 strains from which the whole-genome sequence has been published. Only mutations between inferior strain ER18 and superior strain 16D were considered; hence, additional mutations between the other strains examined were left out of the comparison. The results, summarized in Table 1, show that most mutations found in the superior 16D alleles are not uncommon in other yeast strains. Within the open reading frame of *VMA7*, no differences could be found between the sequences of ER18 and 16D. However, multiple mutations were identified in the promoter, all of which could be found in the other strains examined. The mutations identified in the ORFs of *GLO1*, *HAA1*, *DOT5*, and *CUP2* are commonly found in the other strains. However, the mutation at position c.1517 (G in ER18 to A in 16D) in *HAA1* from 16D was not found in any other strain and may therefore be a novel and unique mutation, possibly important for the superior character of the causative allele in conferring high acetic acid tolerance. This was investigated by introducing only this point mutation into the two copies of the *HAA1* allele of the industrial strain GSE16-T18 (which contains the *HAA1* allele of ethanol red and has been developed for efficient second-generation bioethanol production [27, 33]). Fermentations were performed with YP + 20 % glucose in the presence of 1.0, 1.2, 1.4, 1.6, and 2.0 % acetic acid at pH 5.2 (Fig. 7). This is a relevant sugar density and a relevant pH in second-generation industrial bioethanol production. The strain GSE16-T18 shows already high intrinsic acetic acid tolerance under this condition. In the absence of acetic acid, there was no difference in the fermentation performance between the two strains, while in the presence of all acetic acid concentrations tested the performance of the GSE16-T18 HAA1* strain was consistently better than that of the GSE16-T18 strain. Especially, the lag phase was strongly reduced by the HAA1* mutation, but the actual fermentation rate was also enhanced.

**Table 1:** Occurrence of SNPs in the causative genes *GLO1, HAA1, VMA7, DOT5*, and *CUP2* in a set of 28 yeast strains of which the complete genome sequence is known

	GLO1		HAA1					
	ORF	ORF	ORF	ORF	ORF	ORF	ORF	ORF
	964	106	242	592	694	1025	1091	1130
ER18	A	C	T	G	A	G	TC	T
16D	G	T	C	A	G	A	AT	C
S288C	A	C	C	A	G	A	AT	C
AWRI1631	G	T	C	A	G	A	AT	C
AWRI796	G	T	C	A	G	A	AT	C
BY4741	A	C	C	A	G	A	AT	C
BY4742	A	C	C	A	G	A	AT	C
CBS7960	G	C	C	A	G	A	AT	C
CEN.PK113	A	C	C	A	G	A	AT	C
CLIB215	G	T	C	A	G	A	AT	C
EC1118	G	T	C	A	G	A	AT	C
EC9-8	G	T	C	A	G	A	AT	C
FL100	A	C	C	A	G	A	AT	C
FostersB	G	C	-^a^	–	–	R	WY	Y
FostersO	G	C	–	–	–	A	TC	C
JAY291	G	C	C	G	G	A	TC	C
Kyokai7	A	C	T	G	A	G	TC	T
LalvinQA23	G	–	–	–	–	–	WY	C
PW5	A	C	C	G	A	A	TC	T
RM11-1a	G	T	C	A	G	A	AT	C
Sigma1278b	A	C	C	A	G	A	AT	C
T7	A		T	G	A	G	TC	T
UC5	A	C	C	G	A	G	TC	T
VL3	–	T	–	A	G	A	AT	C
Vin13	G	T	C	R	G	A	WY	C
W303	A	C	C	A	G	A	AT	C
YJM269	A	C	C	G	A	G	TC	T
YJM789	A	C	C	G	A	G	TC	T
YPS163	A	C	C	G	A	G	TC	T
ZTW1	A	T	T	G	A	A	TC	T

			VMA7				
	ORF	ORF	Prom.	Prom.	Prom.	Prom.	Prom.
	1259	1517	−61	−67	−110	−139	−291
ER18	C	G	G	C	G	T	G
16D	A	A*	T	T	C	A	A
S288C	A	G	T	T	C	A	A
AWRI1631	A	G	T	T	C	A	A
AWRI796	A	G	T	T	C	A	A
BY4741	A	G	T	T	C	A	A
BY4742	A	G	T	T	C	A	A
CBS7960	A	G	T	T	C	A	A
CEN.PK113	A	G	T	T	C	A	A
CLIB215	A	G	T	T	C	A	A
EC1118	A	G	T	T	C	A	A
EC9-8	A	G	T	T	C	A	A
FL100	A	G	T	T	C	A	A
FostersB	M	G	T	T	C	A	A
FostersO	C	G	T	T	C	A	A
JAY291	A	G	T	T	C	A	A
Kyokai7	C	G	G	C	G	T	G
LalvinQA23	A	G	C	T	T	A	A
PW5	C	G	T	C	G	A	A
RM11-1a	A	G	T	T	C	A	A
Sigma1278b	A	G	T	T	C	A	A
T7	C	G	T	C	G	A	A
UC5	C	G	G	C	G	T	T
VL3	A	G	T	T	C	A	G
Vin13	A	G	T	T	C	A	A
W303	A	G	T	T	C	A	A
YJM269	C	G	G	C	G	A	A
YJM789	C	G	T	T	C	A	A
YPS163	–	G	T	C	G	A	T
ZTW1	C	G	G	C	G	T	G

	DOT5		CUP2	
	ORF	ORF	ORF	ORF
	25	463	361	497
ER18	G	C	G	T
16D	A	T	A	C
S288C	A	C	A	C
AWRI1631	A	T	A	C
AWRI796	A	C	A	C
BY4741	A	C	A	C
BY4742	A	T	A	C
CBS7960	A	C	A	C
CEN.PK113	A	T	A	C
CLIB215	A	T	A	C
EC1118	A	T	A	C
EC9-8	A	C	A	C
FL100	A	C	A	C
FostersB	A	–	R	C
FostersO	A	T	R	C
JAY291	A	T	A	C
Kyokai7	G	C	G	T
LalvinQA23	A	–	A	C
PW5	A	C	G	T
RM11-1a	A	T	A	C
Sigma1278b	G	C	A	C
T7	A	C	G	T
UC5	A	T	G	T
VL3	G	C	A	C
Vin13	A	T	A	C
W303	A	T	A	C
YJM269	A	C	G	T
YJM789	A	C	A	C
YPS163	A	T	G	T
ZTW1	G	C	G	T

The SNPs present in superior parent 16D compared to inferior parent ER18 were checked in 28 strains of which the whole-genome sequence has been published. SNPs present in the other strains when compared to ER18, but not present in 16D, are not shown. The positions of the SNPs in the ORF or in the promoter are indicated

^a^The dash indicates that no sequence data are available for the specified position

* The *HAA1* allele of strain 16D contains a SNP at position 1517 (A), which is unique compared to the nucleotide (G) at this position in all the other sequenced strains analyzed. This is the only unique SNP present in the superior alleles identified in this work

## Discussion

### Novel genes determining the complex trait of acetic acid tolerance

In total, five genes were confirmed to play a role in the high acetic acid tolerance of the superior segregant 16D. Of these five genes, *VMA7* (QTL4) and *HAA1* (QTL2) were previously linked to acetic acid tolerance. The latter is a transcriptional activator that is well known to be involved in the response to weak acid stress [31, 34, 35]. Interestingly, a novel mutation at position 1517 (A) in the ORF was found in the *HAA1* allele from 16D, which may be responsible for the superior character of the allele. *VMA7* was linked to acetic acid tolerance through a functional screening of the non-essential gene deletion collection [36]. The other three genes, *GLO1* (QTL1), *CUP2* (QTL3), and *DOT5* (QTL5), have not previously been linked to the acetic acid tolerance phenotype and could therefore be novel targets for improving acetic acid tolerance of yeast. The strong genetic linkage of QTL1 suggests that *GLO1* plays a major role in the very high acetic acid tolerance of 16D and its original diploid parent strain JT22689. The gene encodes glyoxylase I, an enzyme responsible for the detoxification of methylglyoxal, a side-product of the triose phosphate isomerase reaction in glycolysis [37]. This suggests that acetic acid stress may enhance the production of methylglyoxal. The *GLO1* gene is induced by osmotic stress in a Hog1-MAP kinase-dependent manner [38]. Interestingly, Hog1-MAP kinase activation is required for acetic acid resistance. The MAP kinase targets the *FPS1* aquaglyceroporin for endocytosis, rendering yeast cells resistant to acetic acid [39, 40]. *GLO1* may therefore present another target of the Hog1-MAP kinase in response to acetic acid stress in yeast.

*DOT5* encodes a nuclear thiol peroxidase which was shown to play a role in oxidative stress resistance. This may provide a link to acetic acid tolerance as this weak organic acid is known to cause oxidative stress [41]. *CUP2* codes for a copper-binding transcription factor that activates metallothionein genes in response to elevated copper levels [42]. Interestingly, *CUP2* is a paralog of *HAA1* [43], which raises the possibility that the *CUP2* allele has taken over the function of *HAA1* in the most superior F7 segregants, explaining why the linkage with *HAA1* was lost after the inbreeding. Since it has previously been reported that *CUP2* and *HAA1* do not exhibit complementing activities [43], mutations in the *CUP2* coding sequence may have altered the conformation of Cup2 into a structure more similar to that of Haa1, allowing it to function also in protection to acetic acid. More detailed analysis of the protein sequences of Haa1 and Cup2 shows that Cup2 is 225 amino acids in length, whereas Haa1 is 694 amino acids. However, alignment of the protein sequences reveals a high degree of similarity in the overlapping part. The first 225 amino acids of Haa1 are 36 % identical and 49 % similar to Cup2. In addition, the highly similar amino acids 1–40 in both proteins were classified as encoding a ‘Copper fist DNA binding domain’ (PFAM domain PF00649).

### Increased recombination frequency generates a different network of causative alleles

Unraveling the complex genetic basis of physiological properties remains a challenge. Especially, the selection of segregants displaying the phenotype of interest can be cumbersome if the trait under study is non-selectable. The development of pooled-segregant whole-genome sequence analysis highly simplified simultaneous genetic mapping of QTLs, a method that works efficiently with a small number of segregants [21–24]. However, identifying causative mutations in the mapped QTLs remains an elaborate task. We have now shown that pooled-segregant whole-genome sequencing analysis can be made more efficient, at least for some QTLs, by inbreeding all segregants multiple times before phenotypic selection. Increasing the recombination frequency resulted in the expected narrowing of QTL size, as was clearly shown for QTL1. However, unexpected outcomes, such as the appearance and disappearance of QTLs, were also observed. The loss of linkage of *HAA1* in QTL2 was particularly striking, since RHA showed that the *HAA1* allele from 16D was truly linked to the superior phenotype and because of the well-established role of *HAA1* in acetic acid tolerance [10, 31]. After multiple inbreeding the linkage of *HAA1* was lost and three other genes, *DOT5, CUP2,* and *VMA7*, were now found and confirmed to be linked to the high acetic acid tolerance of the superior parent strain 16D. QTL1, on the other hand, remained clearly present. This indicates that the selected F7 segregants rely on a partially different set of genes for their high acetic acid tolerance compared to the selected F1 segregants. Moreover, the analysis that took into account the extent of tolerance of the segregants showed that within the F7 population more resistant segregants rely on a different combination of genes than less resistant ones.

The reason for this remains unclear. Increased recombination frequency may uncouple positively and negatively acting genetic elements and may thus generate a new interaction network of causative genes partially different from the previous network. The new network may support the complex trait somewhat better than the previous network, causing these segregants to be ultimately selected for the pool used in mapping. Since the larger genomic fragments originating from the superior parent strain and present in the F1 segregants are more representative of the situation in the superior parent itself, i.e., they contain longer stretches of genes from the original chromosomes than the small fragments in the F7 segregants, the polygenic network elucidated with the F1 segregants may be closest to the real polygenic network present in the superior parent strain. It appears likely that in different strains, polygenic traits can be established by a partially different set of causative genes. The present work shows that such partially different sets of causative genes can also be present within a single strain and that different methodologies can reveal the different sets.

Previous work by Hillenmeyer et al. [44] reported that in some cases, multiple sets of genes can be involved in resistance to the same compound, as was also shown here by the inbreeding strategy. Alternatively, one gene can be linked to tolerance to multiple stresses [44] and such observations were also made in our lab. In earlier papers, we have shown that superior properties like high ethanol tolerance and thermotolerance can be caused by the same gene [21, 25]. In both studies, the gene *MKT1* was identified as causative allele for improved tolerance to high ethanol concentrations and high temperature, showing that indeed one gene can have pleiotropic effects on tolerance to multiple types of stress. On the other hand, no overlap between the causative genes identified for high acetic acid tolerance in the present study and those identified for high ethanol or high thermotolerance in the previous studies was found. Large-scale phenotyping screens revealed also reduced resistance to other types of inhibitors than weak acids, such as cycloheximide and miconazole in the case of *HAA1* [45, 46]. Also for the other genes, deletion mutants turned out to be affected in tolerance to multiple types of inhibitors and/or stress conditions, but the physiological relevance of these high-throughput screening observations has not been established yet [45, 47].

Another observation was the increased number of linked loci found with the F7 segregants in the unselected (control) pool. This is likely a result of the inbreeding strategy, which inadvertently selects for sporulation capacity, spore viability, and mating ability. Since inbreeding crosses can only be performed with sporulating diploids and viable spores that mate well, genes controlling these traits will be inadvertently enriched in the pool of inbred segregants.

### Sequencing of pooled segregants versus individual segregants

Individual sequence analysis with the selected F7 segregants resulted in a higher QTL mapping resolution. By comparing the whole-genome sequence of the 27 selected segregants, we were able to score SNPs and calculate SNP variant frequencies more accurately and use them to calculate the statistical significance of every SNP between ER18 and 16D. In addition to this improvement, we could validate the mapping results obtained with the pooled segregants, as both strategies resulted in similar genetic maps. It was therefore concluded that genetic analysis of complex traits with pooled segregants is still a convenient and efficient approach to simultaneously identify multiple genetic loci, in view of the high cost of sequencing individual segregants. Taking into account their quantitative phenotype, however, enabled discrimination between specific sets of genes enabling tolerance in the most tolerant and less tolerant segregants, providing a more direct insight into the diversity of combinations of genes that constitute a genetic make-up of a certain polygenic trait.

## Conclusions

This work has revealed the polygenic basis of high acetic acid tolerance in *S. cerevisiae* in unprecedented detail, identifying both already known and several novel genes as causative elements. It shows that polygenic analysis of natural variation in yeast strains can reveal novel causative genes involved in industrially important complex traits, in spite of numerous previous omics studies usually performed with laboratory strains aimed at identifying genes underlying such traits. The alleles identified provide novel gene tools to improve the acetic acid tolerance of industrial yeast strains and minimize the risk of side effects often observed with classical genetic engineering techniques. Our work has shown for the first time that a single strain can harbor different sets of causative genes able to establish the same polygenic trait.

## Methods

### Strains and growth conditions

Yeast strains used in this study are shown in Table 2. Yeast cells were grown in a shaking incubator at 30 °C and 200 rpm in YPD medium containing 1 % (w/v) yeast extract, 2 % (w/v) bacto peptone, and 2 % (w/v) d-glucose. Acetic acid medium was prepared by adding acetic acid to YPD medium, after which the pH was adjusted to 4.0 with HCl or KOH. Subsequently, the acetic acid medium was filter sterilized using a 0.2-µm filter. Antibiotics were added as required in the following final concentrations: geneticin, 400 µg ml^−1^; nourseothricin, 100 µg ml^−1^. For solid medium, 1.5 % Bacto agar was added to the YPD medium.

**Table 2:** Strains used in this study

Strain	Description	Source
Ethanol Red	Commercial diploid strain used for first-generation industrial bioethanol production, low acetic acid tolerance	Fermentis
JT22689	Diploid strain, isolated from fermenting must (‘sturm’) in Austria, high acetic acid tolerance	Portuguese yeast culture collection (PYCC 4542)
ER18	Haploid segregant from Ethanol Red with similar acetic acid tolerance, mat**a**	This study
16D	Haploid segregant from JT22689 with similar acetic acid tolerance, mat**α**	This study
ER18 x 16D	Hybrid diploid strain obtained by crossing ER18 and 16D	This study
ER18 x 16D haa1∆	Hybrid diploid strain; ER18 crossed with 16D haa1∆	This study
ER18 haa1∆ x 16D	Hybrid diploid strain; ER18 haa1∆ crossed with 16D	This study
ER18 x 16D dot5∆	Hybrid diploid strain; ER18 crossed with 16D dot5∆	This study
ER18 dot5∆ x 16D	Hybrid diploid strain; ER18 dot5∆ crossed with 16D	This study
ER18 x 16D cup2∆	Hybrid diploid strain; ER18 crossed with 16D cup2∆	This study
ER18 cup2∆ x 16D	Hybrid diploid strain; ER18 cup2∆ crossed with 16D	This study
ER18 x 16D vma7∆	Hybrid diploid strain; ER18 crossed with 16D vma7∆	This study
ER18 vma7∆ x 16D	Hybrid diploid strain; ER18 vma7∆ crossed with 16D	This study
ER18 x 16D ypt7∆	Hybrid diploid strain; ER18 crossed with 16D ypt7∆	This study
ER18 ypt7∆ x 16D	Hybrid diploid strain; ER18 ypt7∆ crossed with 16D	This study
ER18 x 16D glo1∆	Hybrid diploid strain; ER18 crossed with 16D glo1∆	This study
ER18 glo1∆ x 16D	Hybrid diploid strain; ER18 glo1∆ crossed with 16D	This study
ER18 x 16D pma1∆	Hybrid diploid strain; ER18 crossed with 16D pma1∆	This study
ER18 pma1∆ x 16D	Hybrid diploid strain; ER18 pma1∆ crossed with 16D	This study
ER18 x 16D tos3∆	Hybrid diploid strain; ER18 crossed with 16D tos3∆	This study
ER18 tos3∆ x 16D	Hybrid diploid strain; ER18 tos3∆ crossed with 16D	This study
ER18_haa1(16D)	Strain ER18 in which *HAA1* was replaced by superior allele from 16D	This study
ER18 ypt7∆_haa1(16D)	ER18_haa1* carrying ypt7 deletion	This study
GSE16-T18	Industrial yeast strain for second-generation bioethanol production	[27, 33]
GSE16-T18 HAA1*	GSE16-T18 with c.1517 G > A mutation in both copies of *HAA1*	This study

Fermentation experiments were performed in batch under semi-anaerobic conditions in straight glass tubes containing 100 ml YPD medium and various concentrations of acetic acid. The culture was stirred continuously at 120 rpm using a magnetic stirrer. Fermentations were inoculated with 5 ml of a late exponential phase yeast culture in YPD medium (30 °C, static incubation). The progress of the fermentation was monitored by measuring the decrease in weight of the fermentation tube with the yeast cell culture. During fermentation, the glucose in YPD medium is fermented, producing CO_2_ that is emitted from the fermentation tubes. The emission of CO_2_ is reflected by the loss in weight of the fermentation tube.

### Mating, sporulation, and tetrad analysis

Mating, sporulation, and tetrad analysis were performed by standard procedures [48]. The mating type of the segregants was determined by diagnostic PCR for the MAT locus [49].

### Inbreeding crosses

Inbreeding crosses were performed by random spore isolation, followed by mass mating. Random spore isolation was done by resuspending sporulating cells in 25 ml sterile MQ water supplemented with 10 µg ml^−1^ zymolyase, 10 µl β-mercaptoethanol, and glass beads. This cell suspension was incubated overnight in a shaking incubator (200 rpm). It was subsequently vortexed for 5 min, followed by harvesting the spores by centrifugation (5 min, 3000 rpm). The spores were resuspended in 10 ml Nonidet P-40 (1.5 % (v/v) and put on ice for 15 min. After cooling, the suspension was sonicated four times (amplitude = 75 %, cycle = 1) for 30 s with two-min intervals. The suspension was washed three times with Nonidet P-40 and again sonicated four times. Spores were pelleted, resuspended in 300 µl MQ water, and plated in serial dilutions for single colonies. The remaining solution was plated on a single YPD plate and incubated at 30 °C for two nights to allow mass mating of the isolated spores.

### General molecular biology techniques

Genomic DNA was extracted with phenol–chloroform–isoamyl [PCI-alcohol (25:24:1)] as described by Hoffman and Winston [50]. PCR reactions were performed with ExTaq (TAKARA) for diagnostic purposes or Q5^®^ High-Fidelity DNA polymerase (New England Biolabs) for sequencing purposes, both according to manufacturer’s protocols. Yeast was transformed using the LiAc/PEG method [51]. Gene deletions were made using a PCR-based strategy [52, 53]. After transformation, gene deletions were verified by PCR.

### DNA isolation for whole-genome sequence analysis

The two parent strains ER18 and 16D, all 27 segregants displaying high acetic acid tolerance, and 27 randomly picked segregants were individually grown to stationary phase in 10 ml YPD medium. Segregants were pooled, based on OD_600_, such that the number of cells from every segregant in the pool was equal. The genomic DNA was extracted [54]. At least 3 µg of genomic DNA was provided to BGI (Hong Kong) for sequence analysis using the Illumina HiSeq 2000 platform. Paired-end short reads of ~100 base pairs were generated for four samples (ER18, 16D, selected pool and unselected pool). Mapping of the short read sequences, variant calling, and QTL analysis were performed as described previously [21, 23]. The SNP variant frequencies were calculated by dividing the number of the alternative variant by the total number of aligned reads. A very high or a very low frequency was indicative of a one-sided SNP segregation preferentially inherited from one parent, indicating a genetic linkage to the trait of interest. Statistical confirmation of genetic linkage was obtained using NGSEP [30], EXPLoRA [55], and by methods described earlier [21].

### Scoring SNPs by allele-specific PCR

Individual SNPs were scored by PCR using forward and reverse primers that differ only at the 3′ terminal nucleotide, based on the DNA sequence of the gene in ER18 or 16D. The optimal annealing temperature was determined by gradient PCR using DNA of ER18 and 16D. The optimal temperature is the annealing temperature at which only hybridization with primers containing an exact match was observed.

### Bioinformatics analysis of F7 segregants

Paired-end sequencing reads of the parental strains (16D, ER18), pooled F1 and F7 segregants, and individual F7 segregants were quality filtered and aligned to the reference *S. cerevisiae* genome (S288c) using Bowtie (-a -X 1000 –strata –best -m 1). Only uniquely mapped reads were retained for variant calling. For every identified SNP (read coverage ≥15, PHRED quality score ≥35), matched in both parental strains and segregants, the relative distribution of genotypes detected at specific SNPs was calculated in each sample. Kullback–Leibler divergence of genotype distributions between segregant and each of the two parental strains was calculated, and used to determine the prevailing parental strain from which the SNP genotype was most likely inherited. FDR cut-off value 0.007 (i.e., a conservative cut-off value for correcting over 28 samples separately) was used to filter the dataset obtained before proceeding with further analysis. To correct for the sequencing errors/bias, the distribution of the entropy scores along the genome was smoothed using Gaussian filtering function. For each of the analyzed strains, the entropy scores along the genome were evaluated using a sliding window of 3 kb and summing distance values within each interval to determine parent-of-origin linkage information for each locus. Values for all chromosomal loci were set to 1 if determined to be originating from 16D parent, to −1 if originating from ER18 parent, or 0 if parent-of-origin could not be determined. To identify the QTL regions, the data containing parent-of-origin linkage information (1, −1, or 0 for each chromosomal position) was iteratively averaged over the increasing number of individual F7 segregants, starting from the most extremely tolerant and added the less tolerant ones. Averaged values above 0.7 or below −0.7 thresholds were indicative of a non-random SNP segregation at that locus, indicating preferential inheritance from a 16D or ER18 parent, respectively.

### Confirmation of the involvement of mutated alleles in the superior phenotype

Confirmation of the involvement of mutated alleles in the superior phenotype was done by RHA [17]. For RHA, diploid strains were constructed by crossing ER18 and 16D wild-type or derived deletion strains such that the hybrid diploid strain carried only one allele (either from ER18 or 16D) of the candidate gene. Subsequent fermentation experiments were performed with two individual isolates of the constructed diploids.

## Data access

All sequence data have been deposited in the Sequence Read Archive (SRA) at the National Center for Biotechnology Information (NCBI) under the BioProject ID PRJNA275385 with accession number SRP055002.

## Abbreviations

QTL: Quantitative trait locus
QTLs: Quantitative trait loci
YPD: Yeast extract peptone dextrose
SNPs: Single nucleotide polymorphisms
HMM: Hidden Markov model
RHA: Reciprocal hemizygosity analysis
PCI: Phenol chloroform isoamyl alcohol
